# Interspecific interactions facilitate keystone species in a multispecies biofilm that promotes plant growth

**DOI:** 10.1093/ismejo/wrae012

**Published:** 2024-01-31

**Authors:** Nan Yang, Henriette L Røder, Wisnu Adi Wicaksono, Birgit Wassermann, Jakob Russel, Xuanji Li, Joseph Nesme, Gabriele Berg, Søren J Sørensen, Mette Burmølle

**Affiliations:** Section of Microbiology, Department of Biology, University of Copenhagen, Copenhagen 2100, Denmark; Section of Microbiology, Department of Biology, University of Copenhagen, Copenhagen 2100, Denmark; Section for Microbiology and Fermentation, Department of Food Science, University of Copenhagen, Copenhagen 2100, Denmark; Institute of Environmental Biotechnology, Graz University of Technology, Graz 8010, Austria; Institute of Environmental Biotechnology, Graz University of Technology, Graz 8010, Austria; Section of Microbiology, Department of Biology, University of Copenhagen, Copenhagen 2100, Denmark; Section of Microbiology, Department of Biology, University of Copenhagen, Copenhagen 2100, Denmark; Section of Microbiology, Department of Biology, University of Copenhagen, Copenhagen 2100, Denmark; Institute of Environmental Biotechnology, Graz University of Technology, Graz 8010, Austria; Section of Microbiology, Department of Biology, University of Copenhagen, Copenhagen 2100, Denmark; Section of Microbiology, Department of Biology, University of Copenhagen, Copenhagen 2100, Denmark

**Keywords:** interspecies interactions, spatial organization, keystone species, mutualism, multispecies biofilms, plant growth

## Abstract

Microorganisms colonizing plant roots co-exist in complex, spatially structured multispecies biofilm communities. However, little is known about microbial interactions and the underlying spatial organization within biofilm communities established on plant roots. Here, a well-established four-species biofilm model (*Stenotrophomonas rhizophila*, *Paenibacillus amylolyticus*, *Microbacterium oxydans*, and *Xanthomonas retroflexus*, termed as SPMX) was applied to *Arabidopsis* roots to study the impact of multispecies biofilm on plant growth and the community spatial dynamics on the roots. SPMX co-culture notably promoted root development and plant biomass. Co-cultured SPMX increased root colonization and formed multispecies biofilms, structurally different from those formed by monocultures. By combining 16S rRNA gene amplicon sequencing and fluorescence in situ hybridization with confocal laser scanning microscopy, we found that the composition and spatial organization of the four-species biofilm significantly changed over time. Monoculture *P. amylolyticus* colonized plant roots poorly, but its population and root colonization were highly enhanced when residing in the four-species biofilm. Exclusion of *P. amylolyticus* from the community reduced overall biofilm production and root colonization of the three species, resulting in the loss of the plant growth-promoting effects. Combined with spatial analysis, this led to identification of *P. amylolyticus* as a keystone species. Our findings highlight that weak root colonizers may benefit from mutualistic interactions in complex communities and hereby become important keystone species impacting community spatial organization and function. This work expands the knowledge on spatial organization uncovering interspecific interactions in multispecies biofilm communities on plant roots, beneficial for harnessing microbial mutualism promoting plant growth.

## Introduction

Microbe–plant interactions have been extensively studied to apply bacteria as biofertilizers and thereby improve plant performance and health [1–3]. In the past decade, the developments of high-throughput approaches, such as metagenomics, transcriptomics, and metabolomics, have extensively boosted plant microbiome research [1, 4]. However, progress in plant microbiome research is currently hampered by lack of knowledge on the mechanisms shaping microbiome composition and function, especially regarding microbe–microbe interactions [5, 6].

Microbes colonizing plant root surfaces often form a biofilm [1], a spatially structured community where various species co-exist [7]. The proximity of diverse species in biofilms facilitates various interactions such as metabolic cooperation, which determine the community traits and functions ultimately affecting plant health [8, 9]. For example, a recent study used transcriptomic and metabolomic analyses to demonstrate that *Bacillus velezensis* cooperatively interacted with a partner species *Pseudomonas stutzeri* via cross-feeding in a multispecies biofilm, which helped plants alleviate salt stress and promoted plant health [10]. Also, *Pseudomonas fluorescens* interacting positively with *Bacillus licheniformis* in biofilm enhanced plant growth [11]. These studies emphasize the importance of beneficial microbial interactions occurring in biofilm communities interacting with plants. In addition to microbial interactions, biofilm matrix components of *Bacillus subtilis* were also found to stimulate melon growth and resistance against fungal pathogens by affecting the plant lipid metabolism and accumulation of glutathione-related molecules using metabolomics [12]. However, the link between interspecific interactions and spatial organization of such biofilm communities interacting with the host plants is poorly described.

Studying the microscale spatial organization of microbial interactions is instrumental to understanding their potential biological interactions and the role that interactions play in microbial community dynamics and functions [13, 14]. Recently, an increasing number of studies have emphasized the importance of spatial organization in the biofilm community as it reflects the true social interactions influencing relative fitness benefits of individual genotypes [15–17]. For example, spatial segregation was driven by cooperative microbial interactions in *Pseudomonas* and *Arthrobacter* biofilms in porous environments [18]. Besides, spatial organization in *B. subtilis* biofilms, affected by founder cell density, indicated cooperative bacterial interactions [19]. By impacting interspecific interactions, spatial organization can impact community traits and functions. One study found that spatially short-range biotic interactions determined the dynamics and functions of synthetic *Escherichia coli* communities [20]. Similarly, spatial segregation was found under structured conditions balancing the competitive and cooperative interactions to stabilize the community [21]. The current studies on spatial structure, however, largely rest on single- or dual-species communities in artificial model systems and it is less explored how complex multispecies biofilm communities interact and organize spatially in host environments, such as on plant roots.

Networks of interspecies interactions in microbial communities often contain nodes, which represent species with higher numbers of interactions with other species, compared to the average species [22]. Such species are fundamental for community structure and function through these interactions, and their removal may lead to dramatic shifts in community diversity and functioning [23], which is why such species are described as keystone species [24]. Keystone species are mainly identified by community sequencing efforts and co-occurrence patterns; however, as such studies are based on bioinformatic analysis with a lack of strain isolation, experimental validation of “true” keystone species, of which the absence destabilizes the community, is not possible [25]. However, the use of synthetic communities facilitates such “pull-out” validation of keystone species and their role in the community [26].

We used a well-established multispecies consortium composed of *Stenotrophomonas rhizophila*, *Paenibacillus amylolyticus*, *Microbacterium oxydans*, and *Xanthomonas retroflexus* (termed as SPMX), originally co-isolated from a leaf embedded in natural soil [27], in which synergistically enhanced biofilm production was observed [28]. This model community has been recently found to induce plant drought tolerance when co-inoculated *in vivo* in the *Arabidopsis* rhizosphere [29]. In the present study, using the same four-species consortium, we aim to (i) examine whether the SPMX consortium interacts synergistically to form multispecies biofilm on *Arabidopsis* root surface and (ii) study spatial organization and community composition dynamics of the multispecies biofilm, aiming to identify potential keystone species during root colonization and growth. We observed that multispecies biofilm formation promoted plant growth, especially root development. Using a combined approach of 16S rRNA gene amplicon sequencing, fluorescence in situ hybridization and confocal laser scanning microscopy (FISH-CLSM), we identified community colonization patterns and plant promotion unique to the SPMX compared to monospecies inoculation. Combining individual abundances in SPMX with three-dimensional (3D) co-localization and cell aggregate analysis, we identified *P. amylolyticus* as a keystone species, and confirmed loss of plant growth-promoting effects and colonization abilities when *P. amylolyticus* was excluded from the community.

## Materials and methods

### Plant material and growth conditions

*Arabidopsis thaliana* ecotype Columbia-0 (Col-0) was used in this study. The Col-0 seeds were firstly surface-sterilized by rinsing with 1 ml of bleach for 1 min, followed by rinsing with 1 ml of 70% ethanol for 1 min. The seeds were then washed with 1 ml of sterilized water twice after removing the ethanol. The surface-sterilized Col-0 seeds were placed on the Murashige–Skoog (MS) salts medium (Duchefa) containing 1% (w/v) sucrose and 1.5% (w/v) agar, allowing germination. Seeds were firstly vernalized at −4°C for 48 h on the sterile Petri dishes filled with 30 ml MS agar medium per plate, and then cultured in a greenhouse with a photoperiod of 16-h-light/8-h-dark, 150 μmol m^−2^ s^−1^ light intensity, 60% humidity, 23°C for daytime temperature, and 18°C for night. Seven days post germination, seedlings with similar growth phenotype were selected and transferred to the new MS agar plates without addition of sucrose, and were inoculated with a bacterial suspension, and incubated under the same conditions in the greenhouse.

### Bacterial strains and growth conditions

The inoculated four-species consortium consists of *Stenotrophomonas rhizophila* (Sr), *Paenibacillus amylolyticus* (Pa), *Microbacterium oxydans* (Mo), and *Xanthomonas retroflexus* (Xr), termed SPMX. These four strains were originally co-isolated from a leaf embedded in a natural soil environment [27]. The four strains stored as frozen glycerol stocks were streaked on the solid tryptic soy broth (TSB) agar (Sigma, St. Louis, USA) plates. Plates were incubated at 24°C for 48 h, before picking single colonies of each strain and inoculating into 5-ml liquid TSB medium. TSB cultures were incubated on an orbital shaker, at 250 rpm and 24°C overnight. Overnight cultures were then inoculated into 100-ml TSB and incubated under the same conditions prior to use.

### Preparation of bacterial suspension and inoculation

Four-species (SPMX) bacterial preparation followed the standard protocol previously described [29]. In brief, bacterial cells from overnight cultures were harvested by centrifugation at 5000 × *g* for 5 min at 4°C, and then washed twice using a PBS buffer before resuspending the pellets in TSB liquid medium. Cell suspension of each species was directly diluted in TSB to an optical density at 600 nm (OD_600_) of 0.2, measured by a spectrophotometer (Eppendorf AG, Germany), to yield ~10^7^ CFU/ ml before use. For SPMX co-culture, each strain suspension after OD_600_ adjustment was mixed in a ratio of 1:1:1:1 (v/v/v/v) for later use. For three-species SMX co-culture, each strain suspension was also mixed in an equal ratio of 1:1:1 (v/v/v) for later use.

### Microbe-plant co-cultivation assay

Microbe-plant co-cultivation was performed as previously described [30] with modifications. Seven-day-old *Arabidopsis* plant seedlings (leaf production stage, previously defined in [31].), grown on MS medium as described above, were transferred to the new MS agar medium without sucrose and inoculated with either four-species (SPMX), three-species (SMX) co-culture, or a mono-culture suspension according to their similar growth phenotype, such as root length and leaf size. At this point, the root length was ~1 cm. Equal 10-μl of bacterial suspension (OD_600_ = 0.2) of either SPMX, SMX co-culture or monocultures was inoculated directly onto the whole root surface by pipetting, before transferring them to the greenhouse. These sample plants were then harvested at three different time points when incubated with SPMX for 5, 10, and 15 days (D5, D10, and D15), accordingly to the 2, 4, and 6 rosette leaves growth stage of *Arabidopsis* early leaf development, respectively [31]. This was end-point analysis. The *Arabidopsis* seedling roots without any SPMX inoculation were used as negative control. At each time point, the main root length and fresh shoot weight of the plants were measured. To evaluate root colonizing ability of the SPMX and SMX on the entire roots, the full-length roots were transferred into Eppendorf tubes with 1 ml phosphate-buffered saline (PBS) and subjected to a standard sonication protocol to remove bacterial cells from roots according to methods previously applied [32, 33], with some modifications. The Eppendorf tubes with roots inside were placed in a sonication bath (model: USC1200THD, output frequency 45 kHz, 180 W, VWR, Ultrasonic Cleaning Baths), sonicated for 30 s at power 9. The colony forming units (CFU/ml) assay was performed on TSB agar medium with the 10-fold dilution series (10^−2^ to 10^−7^) to obtain cell numbers at each time point. Each bacterial treatment contained three independent replicates. The experiments were performed independently at 5, 10, and 15 days (D5, D10, and D15).

### Fluorescent staining and confocal laser scanning microscopy

Plant seedlings grown for 7 days were directly root-inoculated with 10-μl SPMX suspension either coculture or monocultures (OD600 = 0.2) applied by pipetting and cultivated under the same conditions in the greenhouse as described above. Plant seedlings without bacterial inoculation were used as control. The root samples were harvested after five days and visualized by fluorescent staining and CLSM. The roots, containing SPMX co-cultures and monocultures, were firstly fixed in 4% (w/v) paraformaldehyde at room temperature for 15 min, and then stained with SYTO9 dye (Invitrogen) at a dilution of 1:1000 in PBS and 0.1% (g/L) calcofluor white (CFW, Sigma-Aldrich). Biofilm formation on roots was imaged by CLSM (LSM 800, Zeiss) with a Plan-Apochromat 63x/1.4 oil DIC M27. Z-stacks were recorded using Axiocam 503 mono to obtain three-dimensional (3D) images. The maximum excitation and emission wavelengths for SYTO9 and CFW were 485 and 498, 405 and 433 nm, respectively. The middle section (~5 mm) of the roots was mainly investigated. Representative images are shown in results.

### 16S rRNA fluorescence in situ hybridization and confocal laser-scanning microscopy (FISH-CLSM)

FISH was performed as described previously [34–36] with some modifications and optimizations. Roots containing SPMX co-cultures were fixed with 4% (w/v) paraformaldehyde (diluted in PBS) overnight at 4°C prior to FISH application. Post fixation samples were rinsed in PBS, followed by incubation in permeabilization solution containing 1 mg/ml Lysozyme (Cat. No. L6876, Sigma-Aldrich) for 10 min at room temperature and rinsed twice with PBS. Then, samples were dehydrated with a series of ethanol solutions (ethanolic series: 50, 70, 100%) for 3 min each at room temperature. After that, samples were incubated with the hybridization buffer including 0.9 M NaCl, 20 mM Tris/HCl (pH 7.2), 30% (v/v) formamide, 0.01% (w/v) SDS, fluorochrome-labeled oligonucleotide probes at a working concentration of 5 ng/μl at 46°C for 3 h. Samples were then rinsed twice with 1 ml of pre-warmed (48°C) washing buffer containing 20 mM Tris/HCl (pH 7.2), 5 mM Ethylenediaminetetraacetic acid (EDTA, pH 7.2), 102 mM NaCl, followed by incubation in washing buffer for 15 min at 48°C. The washing buffer was gently removed by pipetting at first, and then the root samples were rinsed by pipetting with 1 ml of ice-cold dH_2_O to eliminate residual salt derived from washing buffer. Plant root samples placed on microscope glass slides were air-dried completely at room temperature. The root samples were mounted immediately in VECTASHIELD Antifade Medium (Cat. No. H-1000, Vector Laboratories, Burlingame, California), covered with coverslips and stored at 4°C for later CLSM visualization. All FISH images were captured by CLSM (LSM 800, Zeiss) with a Plan-Apochromat 63x/1.4 oil DIC M27. Z-stacks were recorded using Axiocam 503 mono to obtain 3D images.

Four HPLC-purified oligonucleotide probes, designed specifically for detection of the four species by FISH [34] (Supplementary Table 1), were synthesized commercially (Eurofins Scientific, France). The four probes were 5′ labeled with four different fluorochromes: Cyanine 5 (Cy5), Cyanine 3 (Cy3), 6-car-boxyfluorescein (FAM), and Pacific blue dye (PaBl) for Pa (*P. amylolyticus*), Mo (*M. oxydans*), Sr (*S. rhizophila*), and Xr (*X. retroflexus*), respectively. The probe labeled with PaBl was obtained from Thermo Fisher Scientific (Life Technologies Europe B.V., Nærum, Denmark). The maximum excitation/emission wavelengths and actual emission wavelength ranges for Cy5, Cy3, FAM, and PaBl were detected in the four separate channels using a flexible detector (GaAsP-PMT) (Supplementary Table 2).

### Confocal image processing and quantification analysis

Images of root samples stained with SYTO9 and CFW were acquired with tile settings of 3 × 3 to obtain 1430 × 1430 pixels, corresponding to physical dimensions of 404.63 × 404.63 μm for each image. For each bacterial treatment, three replicates and representative images with z-stacks of 28 slices (z-stack, 0.5 μm per slice) were acquired on both sides of the center of the root (Total 56 slices), corresponding to physical height of 28 μm for each image. Standard FISH-CLSM images were captured with a zoom setting of 0.5 corresponding to physical dimensions of 202.83 × 202.83 μm (1024 × 1024 pixels), with 50 slices (z-stack, 0.5 μm per slice) corresponding to physical height of 25 μm for each image.

For each sample at each time point, three separate and representative images were acquired from the root surfaces positioned at the middle section (within ~5 mm) of *Arabidopsis* plant main roots in vertical direction. The region of interest selected was the representative multispecies biofilm formation where all four species (SPMX) were present. The sampling area with z-stacks covered almost all biofilms formed on the root surface but not close to the edges/surface of glass slide within the given physical dimensions, acquiring a total area of ~9.2 × 10^6^ μm^2^ (per staining image)/2.1 × 10^6^ μm^2^ (per FISH image), which was >1.0 × 10^5^ μm^2^, the minimum requirement for representative data [37]. The captured raw 3D staining images were pre-processed and stitched in the ZEN 2.3 system, using method “stitching”, selecting dimension reference “All by reference” by CFW channel. Afterwards the images were split into two (staining images)/four (FISH images) channels in software Fiji [38]. Thresholds were set for each channel applying “MaxEntropy” algorithm [39] available in Fiji, to transform each pixel for each channel into binary data. For the root autofluorescence falling into the four channels in FISH images, an open-source graphics editor GIMP-2.10 (https://www.gimp.org) was used to remove these fluorescent signals coming from plant root cells, keeping the target signals of all bacterial cells located on the root surface for the downstream quantification analysis. Biomass volume (μm^3^) was defined as a sum of voxels where the single voxel size (0.04 μm^3^ for staining images, 0.02 μm^3^ for FISH images) was multiplied by the number of voxels with bacterial fluorescent signals in all 3D confocal images [34]. Biomass volume quantification was performed using the “R” statistical programming language [40]. The algorithms for absolute bio-volume quantifications were included in the RCon3D package published on GitHub (www.github.com/Russel88/RCon3D version 1.2.6) [41].

### 3D co-localization and cell aggregate analysis

The processed z-stack FISH images were further analyzed for 3D co-localization between pairs of species, as well as cell aggregates formed by each species in all images. Co-localization analysis used in this study aimed to quantify the occupancy of a target species at certain distances from a focal species by calculating the proportion of pixels occupied by the target species at specific distances to a focal pixel of focal species using the following algorithm. Briefly, in this study, 1000 random pixels of the focal channel were selected. Pixels within 51 μm distance from the focal pixel were grouped in distance bins to the nearest odd integer. For each distance bin, the number of target pixels and total pixels was determined (Fig. S1A, see online supplementary material for a colour version of this figure). The entire analysis was run five times to estimate analytic variability. The final proportion of different target species was then the mean from these five runs. A total of nine z-stack FISH images captured from three biological replicates from three different time points (D5, D10, and D15) were used for these calculations, with standard errors estimated from the biological replicates. This algorithm for co-localization in this study was extended to work with 3D FISH images based on the previous algorithm for 2D co-aggregation analysis in *daime* [42].

3D cell aggregate analysis in this study was used to detect aggregates formed by certain species cells via grouping adjacent pixels based on a detecting matrix generated by a numeric vector indicating the range of adjacent pixels to aggregate in x, y, z directions in actual 3D FISH images. In this study, for example, each species was located at first in the corresponding channel of the 3D images. Briefly, a rigorous detecting pixel matrix 3 × 3 × 3 was created by a numeric vector c(3, 3, 3) to strictly group adjacent pixels where target species occupies. The target species pixels located in different pixels can be grouped as one aggregate when they were detected in the adjacent pixel matrix (3 × 3 × 3), whereas nonadjacent species pixels which were not grouped by the matrix (3 × 3 × 3) were regarded as different aggregates (Fig. S1B, see online supplementary material for a colour version of this figure). The size of each detected aggregate was the total volume of grouped adjacent pixels. Afterwards, in this study, a threshold of 10 cubic microns was set for the size of detected aggregates and then counting the number of those larger than 10 μm^3^ at the scale of 202.83 × 202.83 μm (~42 000 μm^2^). A total of nine z-stack FISH images captured from three biological replicates from three different time points (D5, D10, and D15) were processed for this analysis. The algorithms for both 3D co-localization and aggregate analysis were included in the R package RCon3D published on GitHub (www.github.com/Russel88/RCon3D version 1.2.6) [43].

### Genomic DNA extraction and quantitative PCR (qPCR) experiment

After 5-, 10-, and 15-days of co-cultivation with bacteria, plant roots were moved into a 2-ml Eppendorf tube with 500 μl of PBS buffer, subjected to the sonication protocol described above (see in “Microbe-plant co-cultivation assay”). The roots were then removed and the bacterial genomic DNA in the suspension was extracted using the NucleoSpin 96 Soil DNA Isolation Kit (Macherey-Nagel, Düren, DE) following the manufacturer’s protocol. For preparation of a standard used for absolute quantification, the 16S rRNA gene fragments of *E. coli* were amplified by conventional PCR using the universal primers 27F (5′-AGA GTT TGA TCA TGG CTC AG-3′) and 1492R (5’-TAC CTT GTT ACG ACT T-3′). The PCR products were purified using the QIAquick PCR Purification Kit (Qiagen Gmbh, Hilden, Germany). The concentrations were measured by Qubit Fluorometer (Invitrogen, Carlsbad, CA, USA). 16S rRNA gene copy number in standard was calculated based on the assumption that the average molecular weight of a base pair (bp) is 660 Daltons. The following equation was used to determine the number of copies of DNA template: Number of copies = (DNA concentration (ng/μl) × [Avogadro’s number (6.022 × 10^23^ molecules/mol)])/(length of template (bp) × [1 × 10^9^] × 650) (URI Genomics & Sequencing Center). 16S rRNA gene copy numbers in unknown samples were then determined by interpolation from the standard curve using their respective threshold cycle (Ct) values. The Ct value represents the number of PCR amplification cycles needed to produce fluorescence intensity above a pre-defined threshold.

All qPCR reactions were performed with the Real-time PCR System LightCycler 96 (Roche, Switzerland) using a pair of universal primers 341F (5’-CCT ACG GGA GGC AGC AG-3′) and 518R (5′-ATT ACC GCG GCT GG-3′). Each 20 μl reaction contained 6 μl of H_2_O, 10 μl of 2× qPCRBIO SyGreen Mix (PCR Biosystems Inc, USA), 1 μl of 10 μM of each primer, and 2 μl of genomic DNA (either standard or sample). The qPCR programmes were as follows: 95°C for 2 min, 40 cycles of 95°C for 5 s, 60°C to 65°C for 20–30 s, followed by a standard melt analysis referring to instrument instructions. Data was based on nine biological replicates in three independent experiments at each time point.

### 16S rRNA gene amplicon sequencing library preparation

To investigate the composition of species relative abundance in SPMX multispecies biofilm formed on the plant root surfaces, root samples incubated with SPMX co-culture were collected at D5, D10, and D15, and the differences in relative abundance of individual species over time were analyzed using 16S rRNA gene sequencing. Total genomic DNA of the bacteria (SPMX) from root samples was extracted using the NucleoSpin 96 Soil DNA Isolation Kit optimized for epMotion (Macherey-Nagel, Düren, DE) using the epMotion 5575 robotic platform model (Eppendorf) following the manufacturer’s protocol. Sterilized PBS solution was included during DNA extraction as blank extraction control. The hypervariable V3-V4 region of 16S rRNA gene was amplified with 2 μl template DNA, using 5 μl 5 × Phusion buffer HF, 0.5 μl 10 mM dNTPs, 0.25 μl Phusion high-fidelity (HF) DNA Polymerase (Thermo Fisher Scientific, Waltham, MA, USA), 1 μl 10 μM of each primer (the modified broad primers 341F (5’-CCTAYGGGRBGCASCAG-3′) and Uni806R (5’-GGACTACNNGGGTATCTAAT-3′) [44] in a 25 μl PCR reaction volume. The first PCR program included 1 min at 95°C, 30 cycles of 15 s at 95°C, 15 s at 56°C, and 72°C for 30 s, and then 5 min at 72°C. The primers were then barcoded in the second PCR with only 15 cycles. Molecular grade water and mock community were included in the PCR amplification as negative and positive controls, respectively. All final PCR products were purified using Agencourt AMPure XP beads (Beckman Coulter Genomics, MA, USA) with the 96-well magnet stand. The purified second PCR products were normalized by the SequalPrep Normalization Plate (96) kit (Invitrogen Ltd., Paisley, UK) and then pooled in equimolar concentrations. The pooled library was concentrated using the DNA Clean & Concentrator-5 Kit (Zymo Research, Irvine, CA, USA). Concentrations were then determined using the Quant-iT High-Sensitivity DNA Assay Kit (Life Technologies).

### Amplicon sequencing and data processing

Paired-end sequencing of the amplicon library was performed on a MiSeq System (Illumina) with MiSeq reagent kit v3 (2 × 300 bp, Illumina Inc., CA, USA), including 15.0% PhiX as an internal control. Demultiplexing in sample-specific raw fastq files was carried out directly on the MiSeq platform prior to downstream analysis. Quality filtering, trimming, denoising, merging and chimera removal of paired end sequences were performed using the DADA2 algorithm [45] in the open-source program QIIME2 [46] to generate amplicon sequencing variants (ASVs). Prior to further analysis, non-target reads such as *Chloroplast* and *Mitochondria* were removed. Amplicon sequencing yielded in total 850 124 bacterial reads (average: 94458; range; 50 814-130 028). The identification of specific ASVs of each strain in the four species SPMX was performed via mapping successfully with 100% coverage and identity as cutoff against the referred full 16S rRNA gene sequences of the four species in NCBI GenBank database using Blast [47]. These reads identified to four species SPMX were extracted for further analysis after correction by 16S rRNA gene copy numbers in each species (Fig. S2, see online supplementary material for a colour version of this figure). (Accession numbers of the four-species 16S rRNA gene sequences: Xr-JQ890537; Sr-JQ890538; Mo-JQ890539; Pa-JQ890540) [29, 48].

### Statistical analysis

All statistical analyses were performed with the open-source statistical program R (version 4.0.3) [40] in RStudio [49] or GraphPad Prism (version 8) [50]. R packages including “ggplot2” [51], “ggpubr” [52] were mainly used for plotting in this study. Statistical significance in difference between the two groups (SPMX vs. Non-SPMX control) was determined by the Wilcoxon rank-sum test (Wilcoxon test, *P* < .05). Multiple comparisons among SPMX co-culture and monocultures in root colonization and quantitative PCR for three time points were detected by Kruskal–Wallis Test with Benjamini–Hochberg false discovery rate correction (FDR adjusted *P* < .05).

The data processing and statistical analyses for 16S rRNA gene sequencing were carried out using R, mainly in the R package “phyloseq” [53]. Differential relative abundance of each species obtained by 16S rRNA gene sequencing at three different time points were detected using Kruskal–Wallis Test with Benjamini–Hochberg false discovery rate correction (FDR adjusted *P* < .05). The distinct composition of the SPMX multispecies biofilm across three time points (D5, D10, and D15) evaluated by beta-diversity were visualized via principal coordinate analysis (PCoA). Permutation multivariate analysis of variance (PERMANOVA) based on Bray–Curtis distance (R function “adonis” in R package “vegan” [54]) was performed to test the statistical significance between samples collected from three different time points (D5, D10, and D15) in composition difference of SPMX. The explained variance *R*-square (*R*^2^) value indicated the impact of the days post co-cultivation on relative abundance in SPMX colonizing the root. All obtained *P* values were corrected by FDR.

## Results

### Four-species community promoted root development and increased *Arabidopsis* biomass

**Figure 1:**
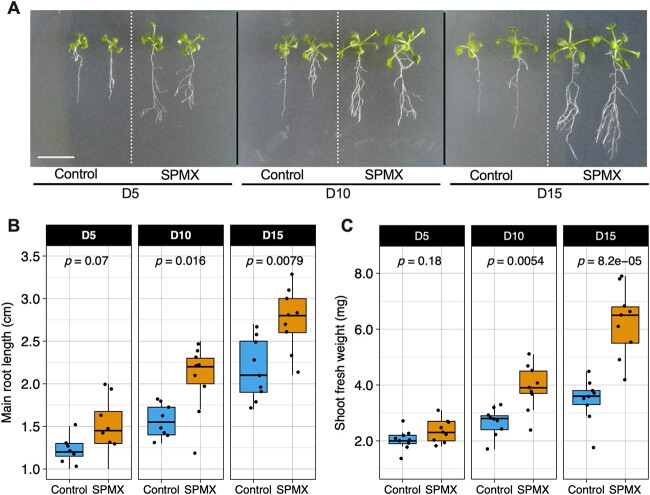
Impacts of SPMX on *Arabidopsis* root growth and shoot fresh weight over time; (A) growth phenotype of seven-day-old *Arabidopsis* seedlings inoculated with SPMX (a four-species consortium composed of *S. rhizophila*, *P. amylolyticus*, *M. oxydans*, and *X. retroflexus*) co-cultured for 5, 10, and 15 days (D5, D10, and D15) (scale bar = 1 cm); (B) boxplot showing main root length (cm) of *Arabidopsis* seedlings incubated with SPMX co-culture at D5, D10, and D15 (*n* = 9); (C) boxplot presenting shoot fresh weight (mg) of *Arabidopsis* seedlings incubated with SPMX co-culture at D5, D10, and D15 (*n* = 9); data in each group are based on nine biological replicates (*n* = 9) from three independent experiments; statistical significance indicates the difference between SPMX-treated and control plants based on Wilcoxon rank-sum test (Wilcoxon test).

To investigate impacts of the four-species (SPMX) community on plant growth, *Arabidopsis* plant seedlings, grown for seven days on MS medium, were inoculated with SPMX co-culture, and incubated over time for 5, 10, and 15 days (D5, D10, and D15). From D5 to D15, the SPMX co-culture remarkably promoted plant growth and root development, including main and lateral roots compared to the growth of control plants without SPMX inoculation (Fig. 1A). At D5, a slight increase was observed in both main root length and shoot fresh weight of SPMX-inoculated plants compared to controls without SPMX inoculation. However, significant growth differences in main root elongation between plants with and without SPMX (control) inoculation were observed at D10 and D15 (median: 2.2 vs. 1.5 cm and 2.8 vs. 2.1 cm; *P* = .016 and *P* = .0079, respectively, Wilcoxon test) (Fig. 1B). Similarly, SPMX inoculation notably increased plant fresh weight to 3.9 mg (median) compared to the control (median: 2.7 mg), corresponding to 44.4% at D10 (*P* = .0054, Wilcoxon test). This significant difference between SPMX-inoculated and non-inoculated control plants was even more pronounced at D15. The shoot fresh weight of plants incubated with SPMX for 15 days was on average increased by 81.1%, compared to control plants (median: 6.7 vs. 3.7 mg, *P* = 8.2e-05, Wilcoxon test) (Fig. 1C). These results showed that the SPMX consortium promoted plant growth and root development with effects lasting at least 15 days.

### Four-species community increased root colonization in *Arabidopsis* compared to individual species

**Figure 2:**
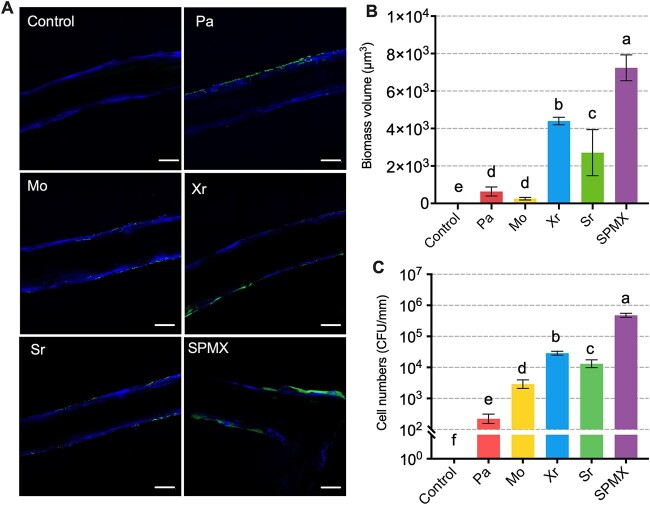
SPMX co-culture and monocultures colonization of *Arabidopsis* roots; (A) representative images captured by confocal laser scanning microscopy (CLSM) showing bacterial colonization of an *Arabidopsis* root after five days (D5) of incubation with SPMX or one of four individual strains of *M. oxydans* (Mo), *S. rhizophila* (Sr), *X. retroflexus* (Xr), and *P. amylolyticus* (Pa); the bacterial colonization was visualized by staining with SYTO9; the root was stained with calcofluor white (CFW) (scale bar = 50 μm); (B) bar chart showing biomass volumes of mixed SPMX and four individual species colonizing the roots quantified by 3D quantitative analysis of confocal images. Letters above bars indicate statistical differences; (C) bar chart showing the number of total bacterial cells that colonize the whole root by SPMX co-culture and monocultures, quantified by colony forming units (CFU) assay and normalized by average root length (mm); letters above bars indicate statistical differences.

To investigate four-species (SPMX) root colonization compared to that of each strain, root samples incubated with SPMX co-culture and mono-cultures for 5 days were visualized by CLSM in combination with SYTO9 and CFW staining. Side roots, containing bacteria (green, stained with SYTO9) distinguished from root cells (blue, stained with CFW) (Fig. 2A). Pronounced bacterial colonization was observed especially on the SPMX-inoculated root, which differed markedly from roots colonized by any individual species (Fig. 2A). Biomass volumes of bacteria colonizing roots were quantified via 3D quantitative analysis of confocal images acquired at D5, comparing differences in root colonization between SPMX co-culture and monocultures. The biomass volume of root colonizing SPMX was significantly higher than that of any of the four individual species (Kruskal–Wallis Test, *P* < .05). Among these, Xr was the best root colonizer, followed by Sr, whereas Pa and Mo exhibited low colonization and poor biofilm formation on the roots (Fig. 2A and B). Almost no fluorescence signals were detected in non-bacteria inoculated controls (Fig. 2B). Similar results were obtained by CFU quantification of total root-colonizing bacterial cells (Fig. 2C), where the bacteria were harvested from whole roots and the count was normalized to the root length (mm). This result suggests enhanced ability of four-species root colonization and biofilm formation on the root surface compared to single species.

### Four-species community established multispecies biofilms on the roots and grew over time

**Figure 3:**
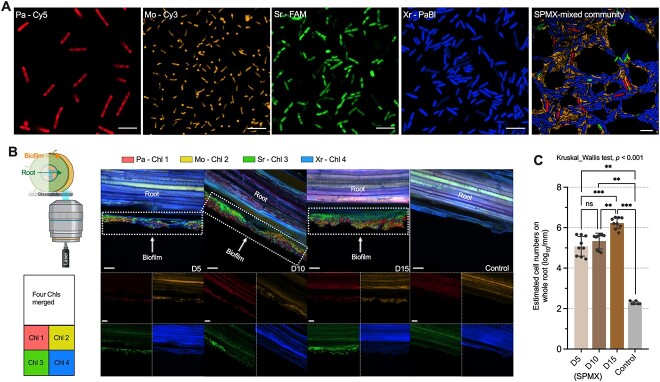
FISH-confocal laser scanning microscopy (FISH-CLSM) micrographs of multispecies biofilm formed by SPMX on the root surface; (A) FISH-CLSM images showing the cell shape and size of each species used in this study, and all four strains (SPMX) in a mixed-species community; the four left images show individual species and one on the far right shows SPMX-mixed community. Pa (*P. amylolyticus*), Mo (*M. oxydans*), Xr (*X. retroflexus*), and Sr (*S. rhizophila*) are labeled with Cy5, Cy3, Pacific blue, and FAM, respectively (scale bar = 5 μm); (B) representative FISH-CLSM images, from SPMX-root co-cultivation samples and non-bacteria inoculated root samples (control), visualizing multispecies biofilm formation over time (D5, D10, and D15), captured from root side of samples; the multispecies biofilm formed on the roots is indicated with a white dashed box and arrow, and the root region is denoted in the images; illustration in the left panel indicates the location of root side where micrographs are captured; micrographs from the root side contain four-channel separate and merged images (scale bar = 10 μm); (C) bar chart showing the estimated total cell numbers in the 5-, 10-, and 15-day multispecies biofilm formed on the whole root (D5, D10, and D15), determined by qPCR and normalized by average root length (mm); data are based on three biological replicates with three root samples in each independent experiment (*n* = 9); Kruskal–Wallis test was used to test the statistical differences of total cell numbers on the roots over time: “ns” refers to “not significant (*P* >.05)”, and ^*^^*^^*^ indicates *P* < .001 (FDR adjusted), ^*^^*^ indicates *P* < .01 (FDR adjusted).

To further analyze the composition and spatial organization of the SPMX multispecies biofilm formed on the roots, FISH was employed to label the four different species in the SPMX biofilm community. Pa, Mo, Sr, and Xr were labeled by Cy5 (red), Cy3 (yellow), FAM (green), and Pacific blue (blue), respectively (Fig. 3A and Supplementary Table 1). Firstly, we confirmed the four oligonucleotide probes specific for the four individual strains by hybridizing SPMX co-culture and four monocultures with all four FISH probes, which indicated the FISH specificity and high hybridization efficiency of the four designed probes (Fig. 3A and Fig. S3, see online supplementary material for a colour version of this figure). A representative four-species (SPMX) biofilm formation, observed at microscale on the root surface, indicated a successful application of the four FISH probes (Fig. S4, see online supplementary material for a colour version of this figure).

The spatial structure of multispecies biofilm formed by SPMX co-cultures on the rhizoplane (root surface) was visualized by FISH-CLSM at D5, D10, and D15 (Fig. 3B and Fig. S5, see online supplementary material for a colour version of this figure). All four species were observed to colonize the root starting from D5, indicating that they all colonized the root at an early stage, whereas no signal derived from bacteria was detected in untreated control roots (Fig. 3B and Fig. S5, see online supplementary material for a colour version of this figure). There was a clear growth of the SPMX multispecies biofilm on the root from D10 to D15, as SPMX was observed to form a relatively thick biofilm on the root compared to that at D5 (Fig. 3B). These observations were supported by qPCR quantification for the total cell enumeration of all four species colonizing the whole roots (Fig 3C). The multispecies biofilm cell numbers increased slightly from D5 to D10 despite no statistical difference (adjusted *P* > .05, Kruskal–Wallis Test) (Fig. 3C). However, the bacterial cell numbers significantly increased from D10 to D15 (adjusted *P* < .01, Kruskal–Wallis Test), which suggested a significant growth of the multispecies biofilm on the root over time. In particular, the root colonization of species Pa was enhanced at D10 and D15, compared to that at D5 (Fig. 3B). Only a low number of 16S rRNA gene copies (~10^2^ per mm) were detected in controls by qPCR (Fig. 3C).

### Four-species community composition significantly changed over time on roots

**Figure 4:**
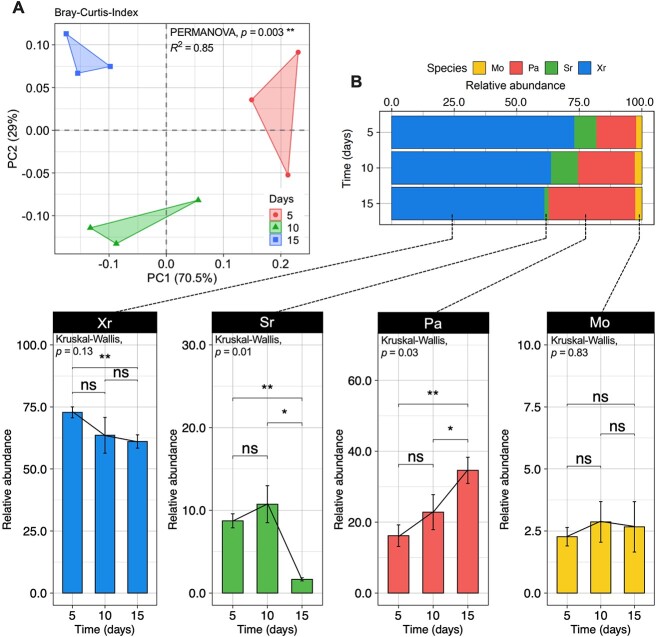
Relative abundance of each species in a four-species biofilm formed on roots over time; (A) principal coordinate analysis (PCoA) showing global differences in composition of the four-species community (SPMX) composed of *M. oxydans* (Mo), *S. rhizophila* (Sr), *X. retroflexus* (Xr), and *P. amylolyticus* (Pa), generated by using the Bray-Curtis distance based on the four species abundance across the three different time points (*n* = 3 for each time point; PERMANOVA, correlation coefficient *R^2^* = 0.85, *P* = .003); the community composition is estimated by 16S rRNA gene sequencing data; (B) bar chart showing differential composition of relative abundance in four species colonizing the *Arabidopsis* root, and abundant shifts of each species over time at D5, D10, and D15 (*n* = 3) based on 16S rRNA gene sequencing data; Kruskal–Wallis test was used to test the statistical differences in relative abundance among three different time points: “ns” refers to “not significant (*P* >.05)”, and ^*^^*^ indicates *P* < .01 (FDR adjusted), ^*^ indicates *P* < .05 (FDR adjusted).

To investigate the community composition and temporal dynamics of SPMX biofilm community on plant roots, *Arabidopsis* root samples incubated with SPMX were collected at D5, D10, and D15. The differences in relative abundance of individual species over time were analyzed with 16S rRNA gene sequencing. At first, the overall difference in composition of the SPMX multispecies biofilm across the three time points (D5, D10, and D15) was evaluated via PCoA. The result indicated that the relative abundances of SPMX biofilm differed from each other (PERMANOVA, *R^2^* = 0.85, *P* = .003) at these three time points (D5, D10, and D15) (Fig. 4A). Furthermore, we analyzed species specific changes in relative abundance in the SPMX multispecies biofilm over time. Overall, Xr was the dominant species in the four-species biofilm at all three time points (D5, D10, and D15), with relative abundance exceeding 50%, followed by Pa, Sr, and Mo (Fig. 4B). However, relative abundance of species Xr decreased from 72.8% to 61.0% at D15 (Kruskal–Wallis Test, FDR adjusted *P* = .13). Similarly, Sr also experienced a drop from 8.7% at D5 to 1.7% at D15 (Kruskal–Wallis Test, FDR adjusted *P* = .01), despite a slight rise to 10.7% at D10. Mo was consistently the least abundant species and did not change much over time (around 2.6% from D5 to D15, Kruskal–Wallis Test, FDR adjusted *P* = .83). In contrast, species Pa in the four-species biofilm showed a significant increase from 16.2% at D5 to 34.6% at D15 (Kruskal–Wallis Test, FDR adjusted *P* = .03), which was consistent with the FISH-CLSM observations on the root surface, where the abundance of Pa increased from D5 to D15 (Fig. 3B). These results show that the relative abundance of the four species in the multispecies biofilm shifted significantly on the roots over time.

### *P. amylolyticus* affected the spatial organization and function of the four-species biofilm community on the roots

**Figure 5:**
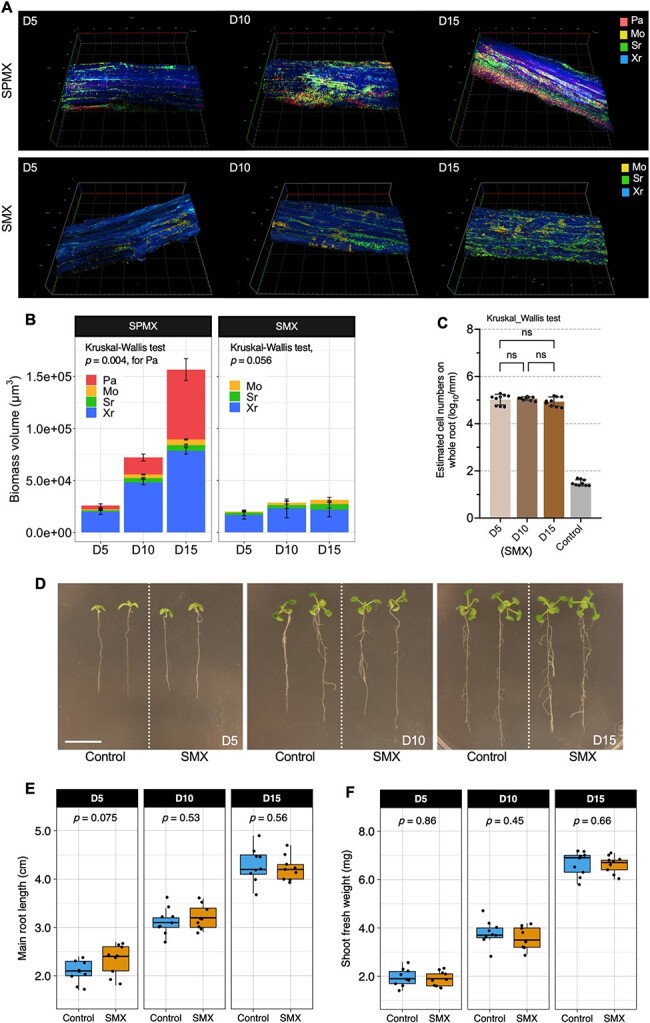
Differences in spatial organization and function between four- and three-species biofilm communities on plant roots; (A) three-dimensional (3D) view displaying the structure of the four-species (SPMX), three-species (SMX) biofilm community established by *M. oxydans* (Mo), *S. rhizophila* (Sr), *X. retroflexus* (Xr) with and without *P. amylolyticus* (Pa) on root surfaces at 5-, 10-, and 15-day post inoculation (D5, D10, and D15), captured by FISH-CLSM with z-stacks; (B) quantification (μm^3^, biomass volume) of each species throughout the SPMX and SMX biofilm at D5, D10, and D15; Kruskal–Wallis test was used to test the statistical difference of the bio-volume of Pa growing at D5, D10, and D15; *P* = .004 in SPMX treatment (FDR adjusted) (*n* = 3); *P* = .056 in SMX treatment (FDR adjusted) (*n* = 6); (C) bar chart showing the estimated total cell numbers in three-species (SMX) biofilm formed on the whole root over time (D5, D10, and D15), determined by qPCR and normalized by average root length (mm) (*n* = 9); Kruskal–Wallis test was used to test the total cell numbers on the root over time: “ns” refers to “not significant (*P* >.05)”; (D) growth phenotype of 7-day-old *Arabidopsis* seedlings inoculated with SMX (a three-species consortium composed of *S. rhizophila*, *M. oxydans*, and *X. retroflexus* in the absence of *P. amylolyticus*), incubated for 5, 10, and 15 days (D5, D10, and D15) (scale bar = 1 cm); (E) boxplot showing main root length (cm) of *Arabidopsis* seedlings incubated with SMX co-culture at D5, D10, and D15 (*n* = 9); (F) boxplot presenting shoot fresh weight (mg) of *Arabidopsis* seedlings incubated with SMX co-culture at D5, D10, and D15 (*n* = 9); data in each group are based on nine biological replicates (*n* = 9) from three independent experiments; statistical significance indicates the difference between SMX-treated and control plants based on Wilcoxon rank-sum test (Wilcoxon test).

To further study the spatial organization of the four-species biofilm community, we investigated spatial dynamics of SPMX biofilm community on the *Arabidopsis* roots at D5, D10, and D15, based on the pixel quantification and analysis of 3D images acquired from FISH-CLSM (Fig. 5A). Firstly, we quantified the absolute abundance (biomass volume) of each species in the SPMX biofilm community on the roots over time. The total bio-volume of SPMX biofilm increased significantly from D5 (~2.5 × 10^4^ μm^3^) to D15 (~1.6 × 10^5^ μm^3^) on the root surface (Fig. 5B). Specifically, it was clear that Pa increased notably and reached a higher abundance in the SPMX biofilm community at D15 compared to Mo and Sr both remaining low in abundance over time (Fig. 5B). Sections of the four-species biofilms through the root, acquired at the three time points, indicated distinct spatial organization of the individual species, where species Sr was frequently positioned at the bottom of the biofilm, near the root (Fig. S6, see online supplementary material for a colour version of this figure). This colonization pattern of Sr was also observed from FISH-CLSM for the root side (Fig. 3B), where Sr was often positioned at the interface between biofilm and root surface. Although the absolute bio-volume of Xr also increased from D5 to D15, the Pa population grew significantly and became a dominant species at D15 (Kruskal–Wallis test, *P* = .004, FDR adjusted). The significant increase of Pa observed in both relative and absolute abundances indicated that Pa might interact mutualistically with the one or all of three other biofilm members (Figs 4B and 5B; Fig. S7, see online supplementary material for a colour version of this figure). This led us to further explore the role of Pa in the community.

To assess the importance of Pa presence, we investigated the spatial organization and colonization of the three-species (SMX) community with exclusion of Pa on the root surfaces. This Pa pull-out significantly affected the thickness and composition in the SMX biofilms on the root surfaces over time, causing significant decreases in bio-volume and root colonization compared to the SPMX community (Fig. 5A). Neither the community composition nor the total biovolume of the SMX community changed significantly over time (Kruskal–Wallis Test, *P* = .056), indicating that the SMX did not grow on the roots, which is markedly different from the SPMX community (Fig 5B). This was supported by qPCR quantification of total bacteria colonizing the whole roots where the colonizing cells did not increase in numbers over time either (Fig. 5C). Moreover, the SMX community (with the exclusion of Pa) lost its growth-promoting effects on root development and biomass production over time (Fig. 5D), as no significant differences were observed between control and SMX-treated plants (Fig. 5E and F). These results suggest that Pa acts a keystone species, affecting the community composition and function in the four-species biofilm community on plant roots.

**Figure 6:**
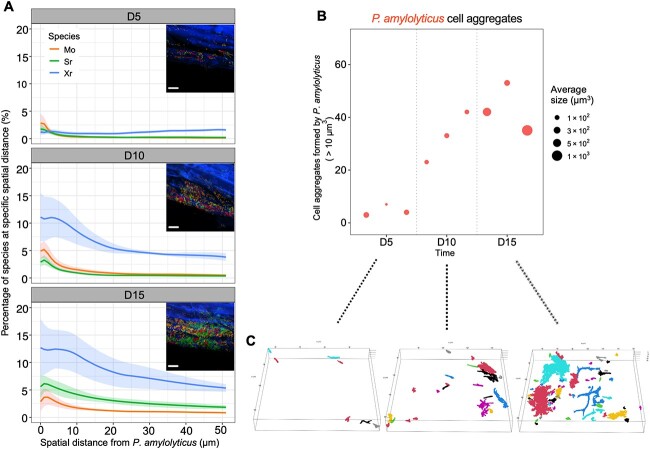
Co-localization and cell aggregate analysis of the multispecies biofilm community on the root surface; (A) co-localization of three biofilm members *M. oxydans* (Mo), *S. rhizophila* (Sr), and *X. retroflexus* (Xr) relative to *P. amylolyticus* (Pa) at D5, D10, and D15 with corresponding observations in magnified confocal images (bar = 10 μm); solid lines are means and shaded regions are SEM of the biological replicates, each with five times repeated analysis; Inserted images show representative results analyzed by 3D co-localization at three time points; (B) microscale cell aggregates of Pa in SPMX multispecies biofilm forming on the roots over time were analyzed by joining neighboring pixels in aggregates at the scale of 202.83 × 202.83 μm, with those larger than 10 μm^3^; point sizes are scaled by average aggregate volume and each point corresponds to one biological replicate; data from three biological replicates at each time point; (C) 3D-view images display simulated Pa cell aggregates identified as larger than 10 μm^3^ on the root surfaces, corresponding to the three time points (D5, D10, and D15); colors of the aggregates are recycled and indicate all different aggregates formed by Pa at the scale of 202.83 × 202.83 μm.

To further investigate how Pa organized spatially to interact with the other species, we analyzed the spatial organization of Pa in relation to the other three species in the biofilm. Firstly, 3D co-localization analysis between Pa and other species were performed to quantify the bio-volume abundance of Mo, Sr, and Xr at certain distances from Pa. Co-localization analysis of Pa with the other three species in the biofilm community showed that the other three species at D5 had low abundances around Pa and seemed to be evenly distributed, although Mo was closer to Pa than both Sr and Xr within 5 μm distance, whereas all three species (Mo, Sr, and Xr) were consistently closely associated with Pa within ~10 μm spatial distance starting from D10 to D15 (Fig. 6A). All three species reached maximum abundances around Pa within ~5 μm spatial distance, whereas the abundances of these species decreased when distances were larger 10 μm from Pa (Fig. 6A). From D10 to D15, the abundance of Xr colonizing around Pa was highest. In addition, although the abundances of all three species in the vicinity of Pa gradually increased from D5 to D15, Sr abundance became higher than that of Mo at D15 compared to D10 (Fig. 6A and inserted images). These results revealed a spatial interaction pattern where the other three biofilm members were enriched around *P. amylolyticus* at ~10 μm microscales.

We next analyzed how each species in the four-species community colonized spatially on the root surfaces via quantifying their cell aggregates over time on roots. At D5, Pa aggregates on the root surface were small and low in numbers whereas at D10, five-time more Pa aggregates (mean of aggregate numbers, 35 vs 7) were present (Fig. 6B). The size of these cell aggregates remained unchanged at D10 compared to D5. However, at D15 the numbers of Pa aggregates had not only increased, but aggregates were also larger. These Pa aggregates at D15 were ~10-fold larger than that at D5 (aggregate size, 10^3^ μm^3^ vs 10^2^ μm^3^) (Fig. 6B), also shown in 3D-stimulated images for different cell aggregates formed by Pa on the root surfaces (Fig. 6C). Such a colonization pattern was not observed in the other species (Mo, Sr, and Xr), although the cell aggregate size of Sr and Xr did increase at D15 (Fig. S8, see online supplementary material for a colour version of this figure). This showed that the Pa population was enhanced via dispersed colonization of the root surface, followed by aggregate growth leading to increased Pa bio-volume and relative abundances. Overall, these results further indicated Pa as a keystone species that impacted the spatial organization and function of the four-species biofilm community on the plant roots.

## Discussion

Root-associated bacteria physically interact with each other to form complex multispecies biofilms on root surfaces, which have a profound influence on plant health and productivity [55]. In the present work, we presented plant growth-promoting effects as an emergent property of a four-species bacterial community (*Stenotrophomonas rhizophila*, *Xanthomonas retroflexus*, *Microbacterium oxydans*, and *Paenibacillus amylolyticus*, SPMX) with biofilm synergy when co-cultured on *Arabidopsis* roots (Fig. 1). The beneficial effects were long-lasting (15 days) and associated with increasing SPMX multispecies biofilm formation. Biofilm formation can generate emergent properties to increase bacterial functionalities and environmental fitness compared to those of single, free-living cells [8, 9, 56, 57]. The SPMX co-culture formed more substantial biofilm structures different from those formed by each species in monocultures (Fig. 2). These observations are in line with previous work, where we showed that emergent properties of the SPMX co-culture led to enhanced plant drought tolerance *in vivo* whereas the monocultures failed to provide such protection [29]. Clearly, co-inoculated SPMX improved root colonization compared to monocultures which colonized roots poorly. Combined, our results highlight the importance of emergent properties of multispecies communities and interactions with potential hosts. Spatial organization has been demonstrated to induce and reflect cooperative and competitive interactions, contributing to functions of multispecies communities [58, 59]. Hence, we next focused on visualizing and analyzing the spatial organization and dynamics of the multispecies biofilm community to uncover interspecific interactions occurring on the plant roots.

An increase in SPMX biofilm biomass was observed on the root surface at D10 and D15 using species-specific FISH probes in combination with CLSM of root segments, which was supported by qPCR quantification (Fig. 3). Our 16S rRNA gene amplicon analysis indicated that the ratio between individual members in the SPMX biofilm community significantly changed over time (Fig. 4A). In accordance with our previous *in vitro* studies [28], Xr strongly dominated the four-species biofilm formed on the plant roots and Xr was consistently most abundant during multispecies biofilm formation on the root from D5 to D15 (Fig. 4B). In contrast, Mo was the lowest abundant community member at all time points. In this study, Pa abundance significantly increased over time, it became the second most abundant species on the roots, whereas it showed the lowest root colonizing abilities on the root when introduced individually compared to any other of the three single species (Fig. 2). This indicates that Pa benefits from the presence and activity of the other biofilm community members and depends on these for colonization and subsequent growth. A previous study found that coexisting bacteria isolated from their natural habitats facilitated synergistic interactions in biofilm formation compared to when isolated from different environments, indicating that coexistence most likely leads to more positive interspecific interactions in the community [60]. Furthermore, interactions in multispecies bacterial communities have been characterized as less competitive and more cooperative over time, resulting in increased biomass of one or all member species [16, 61]. In line with this, Pa abundance at the root surface significantly increased at D15 and occupied the top layer of the biofilm community (Fig. 5A; Fig. S6, see online supplementary material for a colour version of this figure). Although such layered biofilm structure can result from either cooperative or competitive interspecific interactions [17, 62], the absolute abundances of the other three species all increased from D5 to D15 (Fig. 5B). This further indicated mutualistic interactions between Pa and the other three species. The exclusion of Pa reduced biofilm production and root colonization of the three-species community over time (Fig. 5A–C) and this consequently resulted in the loss of plant-beneficial effects of the community (Fig. 5C–E). Combined, these results indicate a key role of Pa in the four-species biofilm community, essential for the community function to promote plant growth.

To further explore the role of Pa in the community, we investigated how Pa organized spatially, to reflect potential interactions with the three other biofilm members on the plant roots by performing microscale 3D co-localization analysis of Pa relative to the other three members, Mo, Sr, and Xr (Fig. 6A). We found that all three members were enriched in the vicinity of Pa, reflected in their enhanced abundances within a short-range spatial distance (< 10 μm) to Pa compared with larger scale distance (> 10 μm), where the abundance of the three species was reduced. This indicates that Pa affected and interacted with those neighboring cells located within 10 μm, potentially facilitating metabolic interactions, which led to biovolume enrichments of all three species within the vicinity of Pa. Metabolic interactions between receiver and producer cells have previously been shown to occur only at the μm range (< 15 μm) [63], supporting our findings of mutual growth benefits of interacting species within a 10 μm range. Earlier research has also found that microbial interactions often emerge in spatially structured settings, where cells only interact with other cells in their proximity [20, 64, 65]. As a consequence of the inter-species interactions with the other three species over time, more Pa aggregates were formed, and the bio-volume of these were also larger (Fig. 6B and C). Such spatial organization was not observed for any of the three other biofilm members (Fig. S8, see online supplementary material for a colour version of this figure), further indicating that Pa as a dominant species shaped the spatial structure and function of this biofilm community. The patchy organization dominated by Pa population could promote social interactions, signaling transduction, and metabolic cooperation in the community, consequently resulting in co-evolution of interspecific cooperation determining community functions to promote plant growth [15]. Also, previous work, conducted under *in vitro* conditions, has proved that high population density can favor evolution of metabolic cooperation in spatially structured environments [66]. Our present findings confirm that the role and function of taxa is highly determined by the ecological interactions in microbial communities, which is also supported by other studies [67]. However, our current findings are not sufficient to defer whether interspecies interactions dictate spatial organization or reverse. To identify such causalities, further work would need to either manipulate spatial organization by such as separating cells by membranes and 3D printing in immobilizing gels, or manipulate interactions by such as preventing metabolic cross-feeding, and subsequently study the effects.

Although Pa in isolation colonized roots poorly, in co-culture it significantly affected the spatial organization and functionality of the four-species biofilm on the roots. We therefore consider Pa as a keystone species on plant roots, according to previous definitions [24], with positive impact on the other community members. This is based on the following findings: (i) enhanced Pa biomass via increasing cell aggregates in both numbers and size over time; (ii) enrichment of all three biofilm members in the proximity of Pa, facilitating potential metabolic cooperation at microscales (~10 μm range); (iii) enhanced absolute abundances of all four community members, despite marked Pa increases in relative abundance, indicating mutualistic interactions; and finally (iv) reduced overall root biofilm production and loss of plant growth-promoting effects by the three-species community (SMX) upon exclusion of Pa. Likewise, such pull-out strategy has successfully identified keystone species in other synthetic communities [68–70]. Further, our findings suggest that a keystone species is not necessarily dominant in the community or possesses strong colonization abilities or functional effects by itself but can establish through mutualistic interactions with other strains and thereby generate emergent properties and potential functions of the community.

Here, we demonstrated the crucial role of Pa in the four-species biofilm community established on the plant roots via high resolution bioimaging and 3D spatial organization analysis, however, more complimentary approaches, such as metabolic modelling [71], transcriptomics [72], and spatial metatranscriptomics [73] in combination with integrated microscale sampling, will be required to further reveal the underlying mechanisms shaping such microbial composition and community function in plant microbiota ecology. A recent study introduced an innovative transcriptome-imaging technique known as parallel sequential FISH (par-seqFISH) for the investigation of *Pseudomonas aeruginosa* in both planktonic and biofilm ecosystems, identifying metabolic heterogeneity at microscopic scale [74]. The tool exhibits remarkable promise for profiling and visualizing the spatial transcriptional and metabolic states at single-cell level, which holds substantial potential for advancing the future studies on microbial ecology, enabling a deeper exploration of the intricate mechanisms governing interspecific interactions within complex microbial communities.

Conclusively, our work presents long-lasting, beneficial effects of a four-species bacterial community on plant growth as a result of the spatial organization of the multispecies biofilms formed on the root surfaces. Our findings unraveled an interspecific interaction pattern that facilitates a keystone species, identified by analysis of spatial organization and dynamics in combination with “pull-out” strategy in the multispecies biofilm community on plant roots. Further, our study demonstrates how a bacterial species, with poor individual root-colonization abilities, establishes and grows, and ultimately becomes a keystone species on roots in the presence of other community members. This is highly relevant in application of bacteria as biofertilizers, as strains promoting and protecting plants may not be strong colonizers by themselves, but able to establish mutualism in communities when co-inoculated with other strains. Thus, this study provides an understanding of spatial organization, uncovering interspecific interactions, shaping the community establishment and function on plant roots, which may benefit future efforts to manipulate the performance of biofertilizers in complex ecological settings [75].

## Supplementary Material

Supplementary_Table_1_wrae012

Supplementary_Table_2_wrae012

FigS1_wrae012

FigS2_wrae012

FigS3_wrae012

FigS4_wrae012

FigS5_wrae012

FigS6_wrae012

FigS7_wrae012

FigS8_wrae012

## Data Availability

The raw sequencing reads for all samples have been deposited in the NCBI Sequence Read Archive (SRA) repository of BioProject database (https://www.ncbi.nlm.nih.gov/bioproject/) with accession number PRJNA997217. The BioSample accession numbers for the amplicon sequences are SAMN36666019-SAMN36666020 under PRJNA997217. The four-species 16S rRNA gene sequences have also been deposited in NCBI GenBank database with accession numbers JQ890537, JQ890538, JQ890539, and JQ890540 for Xr, Sr, Mo, and Pa, respectively. The imaging data have been uploaded to http://mibi.galaxy.bio.ku.dk/Confocal_images_Nan. The script for imaging analysis is available on GitHub (www.github.com/Russel88/RCon3D version 1.2.6).
