# Supplementary material for: Interspecific interactions facilitate keystone species in a multispecies biofilm that promotes plant growth
Source: ISME J. 2024 Jan 31;18(1):wrae012. doi: 10.1093/ismejo/wrae012 (PMC10938371; doi:10.1093/ismejo/wrae012)
Supplement: Supplementary_Table_1_wrae012 [file supplementary_table_1_wrae012.pdf]

Supplementary Table 1. Information of 16S rRNA-directed, fluorochrome-labeled oligonucleotide probes used for FISH in this study.

Probe	Target	Sequence of probe (5'-3')	Fluorochrome	Reference
LGC945	*P. amylolyticus*	CGG TCA GAG GGA TGT CAA GAC	Cy5	This study
HGC118h	*M. oxydans*	CAT GCG TGA AGC CCA AGA C	Cy3	This study
STE448	*S. rhizophila*	CGG GTA TTA GCC GAC TGC TT	FAM	This study
Xan458	*X. retroflexus*	CCG TCA TCC CAA CCA GGT ATT	PaBl	This study
