# Supplementary material for: Interspecific interactions facilitate keystone species in a multispecies biofilm that promotes plant growth
Source: ISME J. 2024 Jan 31;18(1):wrae012. doi: 10.1093/ismejo/wrae012 (PMC10938371; doi:10.1093/ismejo/wrae012)
Supplement: Supplementary_Table_2_wrae012 [file supplementary_table_2_wrae012.pdf]

Supplementary Table 2. List of the maximum excitation/emission wavelengths and actual detection emission wavelength ranges for Cy5, Cy3, FAM, and PaBl in four separate channels.

Channel	Maximum excitation wavelength (nm)	Maximum emission wavelength (nm)	Actual detection emission wavelength range (nm)	Filter
Pa-Cy5	649	670	645-700	No
Mo-Cy3	550	570	400-640	No
Sr-FAM	495	520	410-539	Sp 545
Xr-PaBl	410	455	400-510	No
