# Supplementary material for: Interspecific interactions facilitate keystone species in a multispecies biofilm that promotes plant growth
Source: ISME J. 2024 Jan 31;18(1):wrae012. doi: 10.1093/ismejo/wrae012 (PMC10938371; doi:10.1093/ismejo/wrae012)
Supplement: FigS1_wrae012 [file figs1_wrae012.pdf]

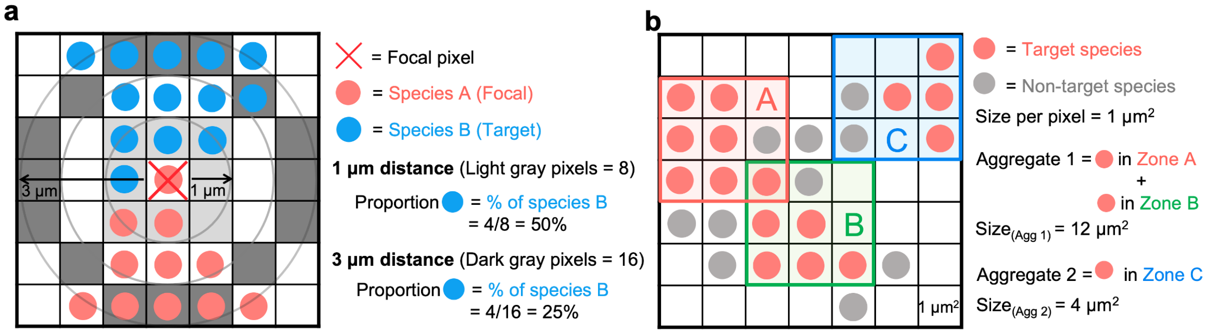

Fig. S1: Schematic diagrams of the 3D co-localization and cell aggregate analysis. The two diagrams are shown in two dimensions (2D), but the actual analysis was performed in three dimensions (3D). **a** Proportion of a target species B (blue) at a certain distance is calculated as the amount of pixels occupied by the target species, divided by the total amount of pixels at that distance from the focal pixel of species A (red) (e.g., distance 1 µm and 3 µm indicated by light and dark gray zones respectively). **b** The cell aggregates formed by the targeted species in red were detected by grouping neighboring pixels based on a detecting pixel matrix generated by c(3, 3, 3) that is a numeric vector indicating range of adjacent pixels to aggregate in the x, y, z directions in actual 3D images. Target species pixels located in Zone A (red) and Zone B (green) were grouped as one aggregate (Aggregate 1) by joining neighboring 3 × 3 pixels. Nonadjacent target species in Zone C (blue) which couldn't be grouped with that in either Zone A or Zone B by joining neighboring 3 × 3 pixel matrix was regarded as the different aggregate (Aggregate 2). Size of each detected aggregate was the total volume of grouped adjacent pixels (Scaling per pixel = 1 µm × 1 µm).
