# Supplementary material for: Interspecific interactions facilitate keystone species in a multispecies biofilm that promotes plant growth
Source: ISME J. 2024 Jan 31;18(1):wrae012. doi: 10.1093/ismejo/wrae012 (PMC10938371; doi:10.1093/ismejo/wrae012)
Supplement: FigS2_wrae012 [file figs2_wrae012.pdf]

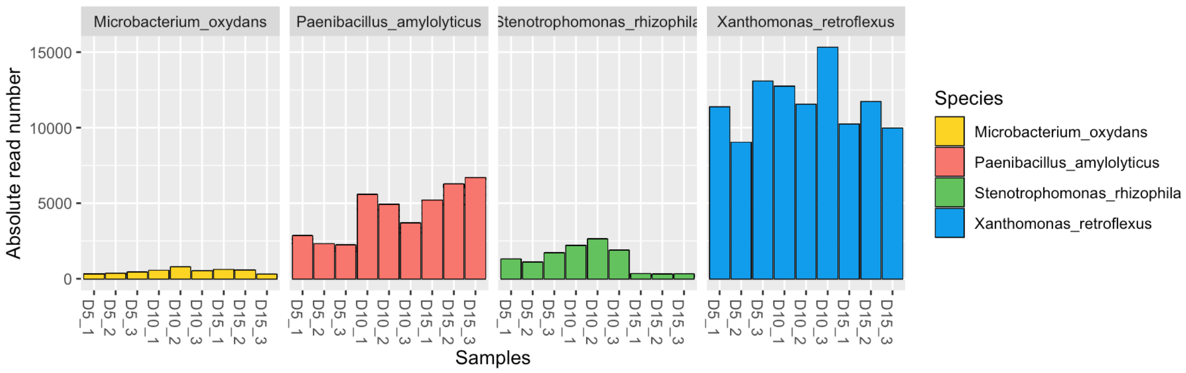

Fig. S2: Absolute read numbers from four species colonizing the plant root surface over time. The bar charts show the absolute read numbers identified into four-species SPMX after corrected by 16S rRNA gene copy numbers in each species at three different time points D5, D10, and D15. Three replicates at each time point (n = 3)
