# Supplementary material for: Interspecific interactions facilitate keystone species in a multispecies biofilm that promotes plant growth
Source: ISME J. 2024 Jan 31;18(1):wrae012. doi: 10.1093/ismejo/wrae012 (PMC10938371; doi:10.1093/ismejo/wrae012)
Supplement: FigS3_wrae012 [file figs3_wrae012.pdf]

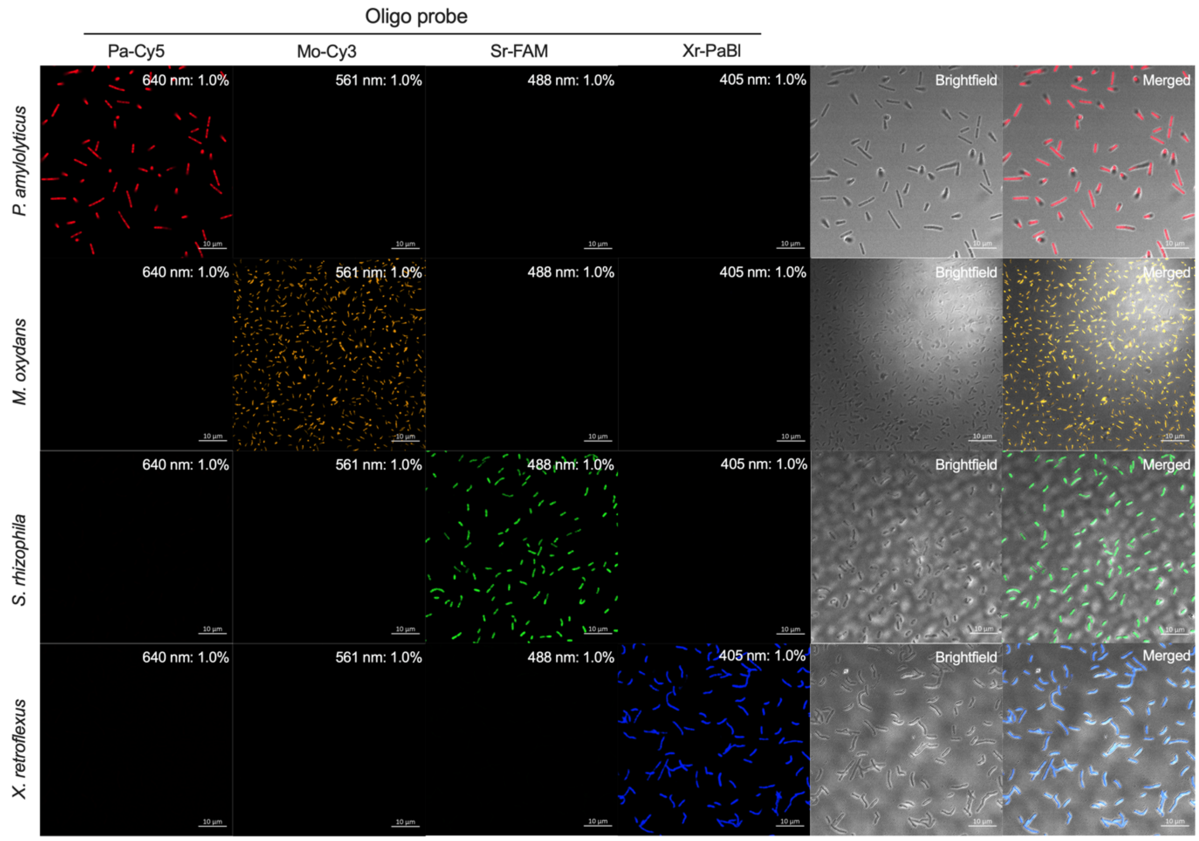

Fig. S3: Validation for four FISH probes specific towards the four species (SPMX) via confocal laser scanning microscopy (FISH-CLSM). One drop of cell suspensions of four individual species (*Paenibacillus amylolyticus*, *Microbacterium oxydans*, *Stenotrophomonas rhizophila*, and *Xanthomonas retroflexus*) was respectively fixed on glass slides and hybridized with hybridization solution containing all four probes at the same time. The laser power for four individual species in each channel (four channels: 405 nm, 488 nm, 561 nm, and 640 nm) was kept as the same of 1.0% to ensure that the fluorescent signal detected in each channel was from specific hybridization of the probe with the target strain. Brightfield and merged images show that cells observed under brightfield in each channel were all covered by the FISH fluorescence, confirming hybridization success. (Scale bar = 10 μm)
