# Supplementary material for: Interspecific interactions facilitate keystone species in a multispecies biofilm that promotes plant growth
Source: ISME J. 2024 Jan 31;18(1):wrae012. doi: 10.1093/ismejo/wrae012 (PMC10938371; doi:10.1093/ismejo/wrae012)
Supplement: FigS4_wrae012 [file figs4_wrae012.pdf]

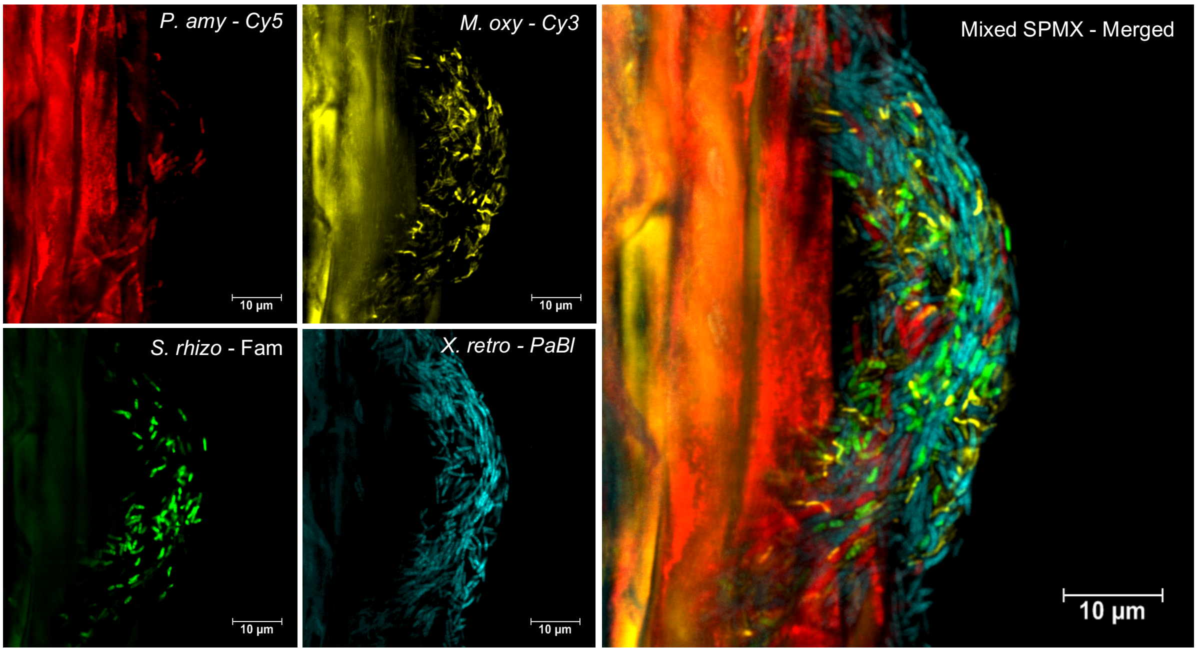

Fig. S4: FISH-Confocal laser scanning (FISH-CLSM) micrographs showing the multispecies biofilm formed by SPMX on the roots. FISH-CLSM images showing the multispecies biofilm composed of four-species SPMX formed on the roots, detected by using four specific FISH oligonucleotide probes. Merged image on the right combined four separated channels on the left. Pa, Mo, Xr, and Sr were labelled with Cy5 (red), Cy3 (yellow), Pacific blue (blue), and FAM (green), respectively. (Scale bar = 10 µm)
