# Supplementary material for: Interspecific interactions facilitate keystone species in a multispecies biofilm that promotes plant growth
Source: ISME J. 2024 Jan 31;18(1):wrae012. doi: 10.1093/ismejo/wrae012 (PMC10938371; doi:10.1093/ismejo/wrae012)
Supplement: FigS5_wrae012 [file figs5_wrae012.pdf]

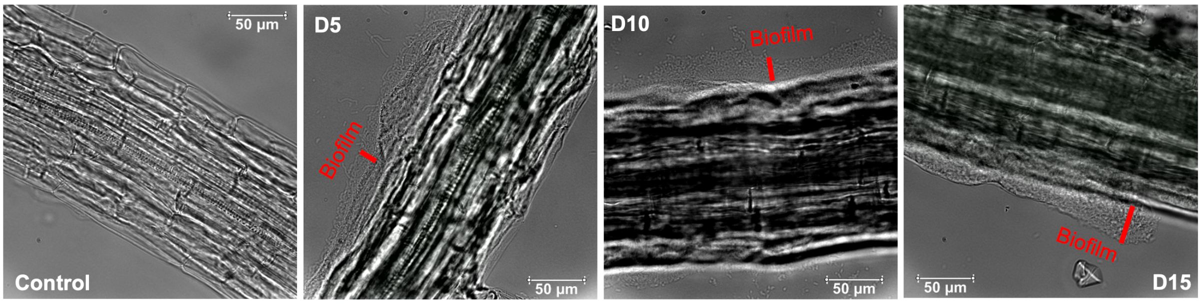

Fig. S5: Confocal laser scanning (CLSM) micrographs showing multispecies biofilm formation by SPMX on the root surface under bright field. CLSM images showing the SPMX multispecies biofilm formed at D5, D10, and D15 on the roots. Non-SPMX inoculated root was used as negative control. The biofilm was denoted by red lines. (Scale bar = 50 µm)
