# Supplementary material for: Interspecific interactions facilitate keystone species in a multispecies biofilm that promotes plant growth
Source: ISME J. 2024 Jan 31;18(1):wrae012. doi: 10.1093/ismejo/wrae012 (PMC10938371; doi:10.1093/ismejo/wrae012)
Supplement: FigS6_wrae012 [file figs6_wrae012.pdf]

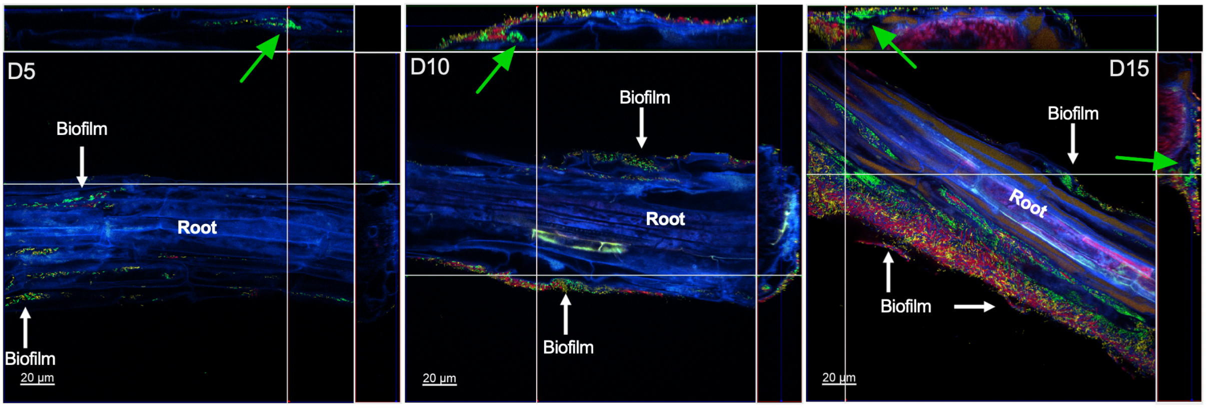

Fig. S6: Orthogonal image displaying the sectioning view of SPMX multispecies biofilm composed of Pa (red), Mo (yellow), Xr (blue), and Sr (green) over time at D5, D10, and D15, captured by FISH-CLSM. The multispecies biofilms formed on the roots are indicated by white arrows and the root region is denoted in the images. Sr located at the bottom of the biofilm is denoted by green arrows (Scale bar = 20 µm).
