# Supplementary material for: Interspecific interactions facilitate keystone species in a multispecies biofilm that promotes plant growth
Source: ISME J. 2024 Jan 31;18(1):wrae012. doi: 10.1093/ismejo/wrae012 (PMC10938371; doi:10.1093/ismejo/wrae012)
Supplement: FigS7_wrae012 [file figs7_wrae012.pdf]

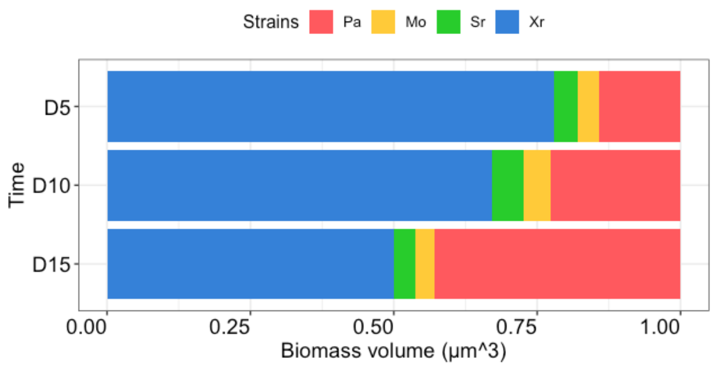

Fig. S7: Composition shifts of species in relative abundance in four-species multispecies biofilm developed on the roots over time. Changes of each species in relative abundance in SPMX biofilm formed on the *Arabidopsis* root from D5 to D15 (n = 9) based on the biomass volumes (μm^3) quantified by 3D confocal images acquired from FISH-CLSM.
