# Supplementary material for: Interspecific interactions facilitate keystone species in a multispecies biofilm that promotes plant growth
Source: ISME J. 2024 Jan 31;18(1):wrae012. doi: 10.1093/ismejo/wrae012 (PMC10938371; doi:10.1093/ismejo/wrae012)
Supplement: FigS8_wrae012 [file figs8_wrae012.pdf]

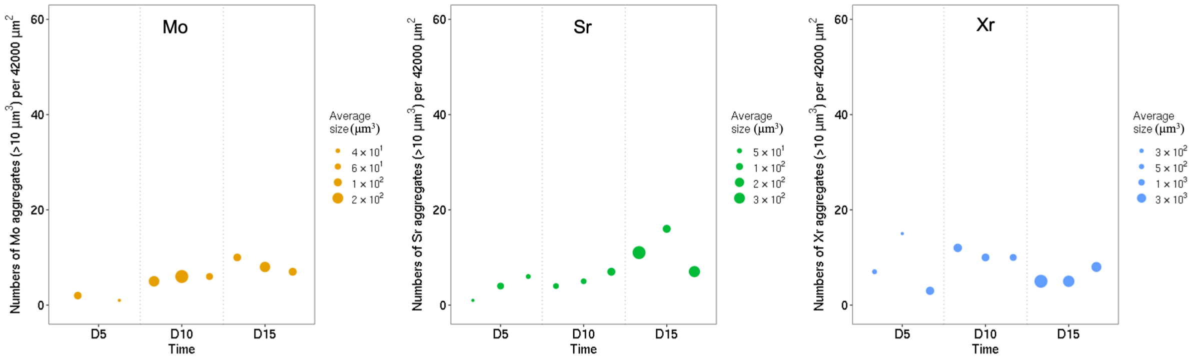

Fig. S8: Microscale cell aggregates formed by species Mo, Sr, and Xr respectively in SPMX multispecies biofilm formed on the roots over time. Microscale aggregates were analyzed by joining neighbouring pixels in aggregates and then counting those aggregates larger than 10 μm^3 at the scale of 202.83 × 202.83 μm. Point sizes are scaled by average aggregate volume (μm^3), each point corresponding to one biological replicate. Data from three biological replicates
